# Screening Characteristics of Bedside Ultrasonography in Confirming Endotracheal Tube Placement; a Diagnostic Accuracy Study

**Published:** 2017-01-09

**Authors:** Hamid Zamani Moghadam, Mohamad Davood Sharifi, Hasan Rajabi, Mojtaba Mousavi Bazaz, Ali Alamdaran, Niazmohammad Jafari, Seyed Amir Masoud Hashemian, Morteza Talebi Deloei

**Affiliations:** 1 Department of Emergency Medicine, Mashhad University of Medical Sciences, Mashhad, Iran.; 2 Department of Social Medicine, School of Medicine, Mashhad University of Medical Sciences, Mashhad, Iran.; 3 Department of Radiology, Mashhad University of Medical Sciences, Mashhad, Iran.

**Keywords:** Ultrasonography, intubation, intratracheal, airway management, emergency service, hospital

## Abstract

**Introduction:**

Confirmation of proper endotracheal tube placement is one of the most important and lifesaving issues of tracheal intubation. The present study was aimed to evaluate the accuracy of tracheal ultrasonography by emergency residents in this regard.

**Method:**

This was a prospective, cross sectional study for evaluating the diagnostic accuracy of ultrasonography in endotracheal tube placement confirmation compared to a combination of 4 clinical confirmation methods of chest and epigastric auscultation, direct laryngoscopy, aspiration of the tube, and pulse oximetry (as reference test).

**Results:**

150 patients with the mean age of 58.52 ± 1.73 years were included (56.6% male). Sensitivity, specificity, positive predictive value, negative predictive value, and positive and negative likelihood ratio of tracheal ultrasonography in endotracheal tube confirmation were 96 (95% CI: 92-99), 88 (95% CI: 62-97), 98 (95% CI: 94-99), 78 (95% CI: 53-93), 64 (95% CI: 16-255), and 0.2 (95% CI: 0.1-0.6), respectively.

**Conclusion:**

The present study showed that tracheal ultrasonography by trained emergency medicine residents had excellent sensitivity (>90%) and good specificity (80-90) for confirming endotracheal tube placement. Therefore, it seems that ultrasonography is a proper screening tool in determining endotracheal tube placement.

## Introduction

In the advanced cardiac life support (ACLS 2010) guidelines, one of the first essential steps in resuscitation is to have a confident airway so that ventilation can be continued properly (1). There are different ways to provide a proper airway for a patient who needs help for breathing. In some situations, like cardiac arrest, respiratory failure and loss of consciousness, only a good intubation can save their life (2). On the other hand, misplacement of the tracheal tube is a life-threatening situation, which leads to a high mortality and morbidity rate. The incidence of esophageal intubation is reported to be about 6-16% in emergency settings (3, 4).

The complications of the tube being placed in esophagus are increasing likelihood of gastric content aspiration into the respiratory system, shortness of breath accompanied by stomach volume expansion with the excess volume of air input, and the most important, losing the golden time to intubate, and subsequent hypoxia of basic organs (5). According to this concept, early detection of misplacement of tracheal tube is important and can be lifesaving.

The clinicians have suggested many different ways to confirm the placement of the tube including traditional methods like direct laryngoscopy to see vocal cords, observation of chest movement, epigastric auscultation, noting the water steam in the tube, and feeling air exiting from the end of the tube after inflation (6). Each of these has limitations that make them not reliable enough to be used as a gold standard for proper placing of the tube in emergency settings, confidently. The second group of methods to confirm the tube placement is para clinical modalities like chest x-ray, pulse–oximetry, capnography, and ultrasonography. Some researchers have introduced quantitative capnography as the most sensitive tool to distinguish tracheal tube placement (7). According to the American Heart Association (AHA 2010) guidelines, quantitative wave waveform capnography is the most reliable method for confirming the tracheal tube placement (1). But, this diagnostic method is not available in every emergency department. In addition, like other methods, it gives some false negative and positive results (8).

Today, ultrasonography is a common tool in emergency physicians’ hand. So it can be proposed as a fast, low–cost and portable method to confirm proper tube placement. Many clinical studies have been carried out to evaluate the accuracy of ultrasonography in correct placement of endotracheal tube (9-21). Some of these studies are cadaveric ones, but most of them are done on patients in emergency settings. However, information regarding feasibility and accuracy of this method particularly when performed by emergency residents is lacking in Iran, since emergency medicine is a young specialty in this country. The present study was aimed to determinate the accuracy of ultrasonography by emergency residents for confirmation of correct tracheal tube placement in real time.

## Methods

***Study design and setting***

This was a prospective, cross sectional study, which was performed in the emergency department (ED) of Imam Reza Hospital, Mashhad, Iran, between March and September 2014, aiming to evaluate the diagnostic accuracy of ultrasonography in endotracheal tube placement confirmation. The study protocol was approved by the committee on medical ethics in research in Mashhad University of Medical Sciences. All subjects were chosen from ED of Imam Reza hospital, which is a tertiary teaching and research hospital. The researchers adhered to the principles of Helsinki Declaration. 

***Participants***

Patients who needed a secure airway due to having no spontaneous respiratory attempt were included in the study using convenience sampling method. These individuals were either intubated primarily on arrival or secondarily after a period of staying in ED. The inclusion criteria involved all the indications of tracheal intubation like failure of ventilation or oxygenation, failure to maintain or protect the airway, and the patient’s anticipated clinical course and likelihood of deterioration. Patients were excluded if they had severe neck trauma, neck masses, a history of any neck operations, and were under the age of 18 years.

***Measurement***

**Figure 1:**
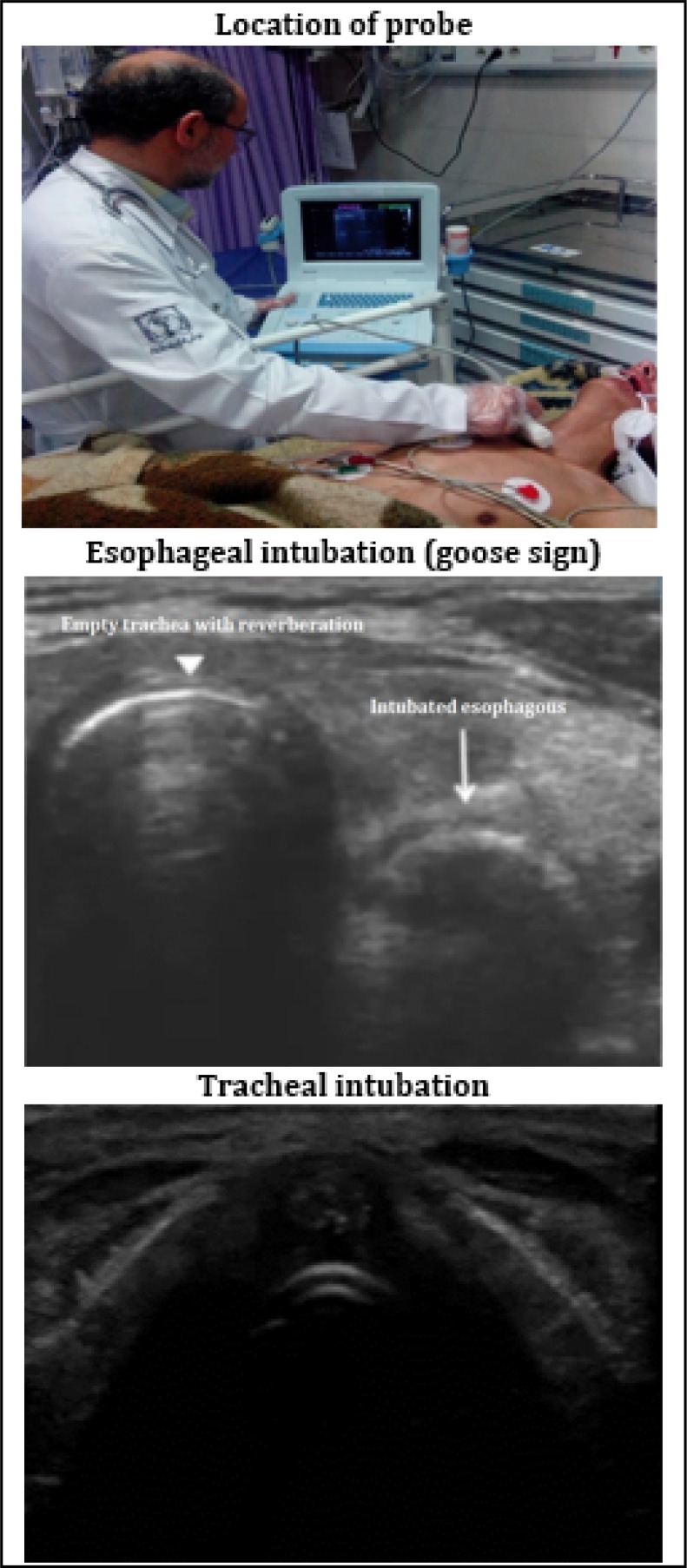
Tracheal ultrasonography.

The intubation process was performed by senior emergency medicine residents. Then another senior emergency medicine resident, which was unaware of the tube placement, checked the tube using ultrasonography (Honda HF-2100, Japan) with a 5.0-7.5 MHz linear transducer. These residents were educated in ultrasonography techniques and how to use it for confirmation of the tracheal tube placement. Simultaneously, another resident confirmed the tube placement using four methods: chest and epigastric auscultation, direct laryngoscopy, aspiration of the tube, and pulse oximetry. If three of these methods showed that the tube is in the trachea, endotracheal intubation was confirmed. These 4 methods together were considered as the reference test and ultrasonography (index test) was compared with their result. Every time intubation and confirmation of the placement were done, an attending emergency physician had been supervising the whole process. Figure 1 shows the location of probe as well as ultrasonographic views of correct and incorrect tube placement. The probe was placed transversely on the neck in lateral position and in front of cricoid cartilage (approximate to sixth cervical vertebra or C6). In a normal anatomy of neck, trachea is seen as a non-compressible circular structure, which is hollow with reverberation. Laterally, the esophagus is seen with smaller size and comprisable lumen, which makes the image of a collapsed donut. However, when the tube is in the esophagus, we can see two circular structures together. The tube in esophagus makes an image of two parallel lines named “goose sign” (Figure 1).

***Statistical analysis***

Previous studies showed that sensitivity of ultrasound in determining endotracheal tube placement was 100% (12). Sample size was calculated to be 131 patients based on a 95% confidence interval, a desired precision of 0.01 and prevalence of 30%. All statistical analyses were performed using SPSS statistical software version 20.0. Evaluating the screening performance characteristics of ultrasonography by residents in conformation of correct tracheal intubation, sensitivity, specificity, positive predictive value (PPV), negative predictive value (NPV), positive likelihood ratio (PLR), negative likelihood ratio (NLR) as well as area under the receiver operating characteristics (ROC) curve with 95% confidence interval (CI) were calculated.

## Results

150 patients with the mean age of 58.52 ± 1.73 years were studied (56% male). The most frequent indications of intubation were loss of consciousness in 63 (42%) cases, respiratory failure in 52 (34.7%), cardiac arrest in 15 (10%), and prophylactic in 20 (13.3%) patients. Based on the findings of the reference test, placement of tube was correct in 133 (88.7%) cases (tracheal intubation) and incorrect in 17 (11.3%) (Esophageal intubation). Emergency resident correctly reported 129 (97%) cases of tracheal intubation (number of true positive) and 15 (88.2%) cases of esophageal ones (number of true negative) using ultrasonography. The overall accuracy of ultrasonography by emergency resident in confirmation of tracheal intubation based on the area under the ROC curve was 92 (83-100). The sensitivity, specificity, PPV and NPV, and PLR and NLR of emergency resident performance of ultrasonography, compared to the combination of 4 clinical methods as the reference test, were 96 (95% CI: 92-99), 88 (95% CI: 62-97), 98 (95% CI: 94-99), 78 (95% CI: 53-93), 64 (95% CI: 16-255), and 0.2 (95% CI: 0.1-0.6), respectively. Screening characteristics of ultrasonography by emergency resident for detecting correct tracheal intubation based on different causes of intubation are shown in table 1.

## Discussion

The present study showed excellent sensitivity and good specificity of ultrasonography by emergency resident in confirming endotracheal tube placement. Ultrasonography is available in almost all emergency departments. Unlike other methods of proper tube placement confirmation, ultrasonography can distinguish the place of tube before bag-valve-mask ventilation, so it can prevent ventilation of stomach and its complications like aspiration. In addition, using ultrasonography does not interrupt the CPR process like other methods do. Therefore, it seems that ultrasonography is a proper screening tool in determining endotracheal tube placement.

**Table 1:** Screening characteristics of ultrasonography in determining endotracheal tube placement based on cause of intubation

**Values**	**Loss of consciousness**	**Respiratory failure**	**Cardiac arrest**	**Other causes**
**Sensitivity**	98.2 (98.2-99.9)	97.9 (87.5-99.9)	84.6 (53.7-97.3)	100 (75.9-100)
**Specificity**	85.7 (42.0-99.2)	100 (39.6-100)	100 (19.9-100)	75.0 (21.9-98.7)
**Accuracy**	96.8 (92.5-100)	98.0 (94.3-100)	86.7 (69.5-100)	95.0 (85.4-100)
**PPV**	98.2 (98.2-99.9)	100 (90.6-100)	100 (67.8-100)	94.1 (96.2-99.7)
**NPV**	85.7 (42.0-99.2)	80.0 (29.9-99.0)	50.0 (9.2-90.8)	100 (30.3-100)
**PLR**	6.9 (1.1-42.2)	NA	NA	4.0 (73.3-21.8)
**NLR**	0.02 (0.002-0.2)	0.02 (0.003-0.1)	0.15 (0.04-0.6)	0.0 (0.0-0.0)
**AUC**	0.92 (0.82-0.97)	0.99 (0.90-1.0)	0.92 (0.68-1.0)	0.88 (0.68-0.99)

PPV: Positive predictive value; NPV: Negative predictive value; PLR: Positive likelihood ratio; NLR: Negative likelihood ratio; AUC: Area under the ROC curve; NA: not applicable due to the calculation cannot be performed because the values include one or more instances of zero.

Many other researchers had been reaching the same results in their studies and concluded that tracheal ultrasound can be a reliable method for assessing placement of tracheal tube in emergency settings. For example, Ma et al. (9) evaluated correctness of tracheal intubation in two phases, dynamic (when intubation was proceeded) and static (after the intubation procedure), by trans-tracheal ultrasonography. They deducted a sensitivity of 97% and specificity of 100% in dynamic phase of intubation but a lower specificity was detected in the static phase (57%). In another study, Chou et al. (14) studied 112 patients. Among them, 15.2% of cases had esophageal intubations and ultrasound could detect a high rate of these displacements by an accuracy of 98.2%, and the sensitivity and specificity of 98.9% and 94.1%, respectively. There are some other researches, which have found 100% sensitivity and specificity for ultrasonography in determining tube placement, but the main limitation of most of these studies is having a small sample size and therefore, they may not show the real characteristics of a large number of people referring to a tertiary hospital in a big city. For example, Werner et al. (11) assessed only 33 patients. On the other hand, there are few studies challenging the diagnostic value of ultrasonography by emergency medicine residents for evaluation of tube placement. For instance, Sim et al. (13) reported a specificity of 55.6% for ultrasonography. 

There are two recent review articles on this topic. The first one included 12 human studies and calculated a sensitivity of 0.93 and specificity of 0.97 with the positive and negative likelihood ratios of 26.98 and 0.08, respectively (22). The other Meta–analysis reviewed 11 articles with 962 intubations in total. They calculated the sensitivity and specificity of 98% for ultrasonography as the confirmation method of tracheal tube placement (23).

***Limitations***

Our study had some limitations. We didn’t estimate the time needed for each method, an important factor in selecting a proper method especially in emergent situations. However, studies have shown that the time needed for ultrasonography is significantly lower than capnography (23, 24). Therefore, it is safe to say evaluation of proper placement of tracheal tube using ultrasonography is faster than capnography. Another limitation of this study was not using capnography as the reference test. Although we tried to reduce performing bias to a minimum by using 4 methods of direct laryngoscopy, clinical auscultation, aspiration and pulse oximetry, misclassification of patients might have affected the accuracy reported in the present study. In addition, convenience sampling in this study is a possible source of publication bias. 

## Conclusion:

The present study showed that tracheal ultrasonography by trained emergency medicine residents had excellent sensitivity (>90%) and good specificity (80-90) for confirming endotracheal tube placement. Therefore, it seems that ultrasonography is a proper screening tool in determining endotracheal tube placement.
